# Finding orthologous gene blocks in bacteria: the computational hardness of the problem and novel methods to address it

**DOI:** 10.1093/bioinformatics/btaa794

**Published:** 2020-12-29

**Authors:** Huy N Nguyen, Alexey Markin, Iddo Friedberg, Oliver Eulenstein

**Affiliations:** Department of Veterinary Microbiology and Preventive Medicine, Iowa State University, Ames, IA 50011, USA; Department of Computer Science, Iowa State University, Ames, IA 50011, USA; Department of Computer Science, Iowa State University, Ames, IA 50011, USA; Department of Veterinary Microbiology and Preventive Medicine, Iowa State University, Ames, IA 50011, USA; Interdepartmental Program in Bioinformatics and Computational Biology, Iowa State University, Ames, IA 50011, USA; Department of Computer Science, Iowa State University, Ames, IA 50011, USA; Interdepartmental Program in Bioinformatics and Computational Biology, Iowa State University, Ames, IA 50011, USA

## Abstract

**Motivation:**

The evolution of complexity is one of the most fascinating and challenging problems in modern biology, and tracing the evolution of complex traits is an open problem. In bacteria, operons and gene blocks provide a model of tractable evolutionary complexity at the genomic level. Gene blocks are structures of co-located genes with related functions, and operons are gene blocks whose genes are co-transcribed on a single mRNA molecule. The genes in operons and gene blocks typically work together in the same system or molecular complex. Previously, we proposed a method that explains the evolution of orthologous gene blocks (orthoblocks) as a combination of a small set of events that take place in vertical evolution from common ancestors. A heuristic method was proposed to solve this problem. However, no study was done to identify the complexity of the problem.

**Results:**

Here, we establish that finding the homologous gene block problem is NP-hard and APX-hard. We have developed a greedy algorithm that runs in polynomial time and guarantees an O(ln⁡n) approximation. In addition, we formalize our problem as an integer linear program problem and solve it using the PuLP package and the standard CPLEX algorithm. Our exploration of several candidate operons reveals that our new method provides more optimal results than the results from the heuristic approach, and is significantly faster.

**Availability and implementation:**

The software and data accompanying this paper are available under the GPLv3 and CC0 license respectively on: https://github.com/nguyenngochuy91/Relevant-Operon.

## 1 Introduction

In bacteria and archaea, gene blocks are sets of genes co-located on the chromosome, which are typically conserved, to some extent, between species. Operons can be viewed as a special case of gene blocks where genes are co-transcribed as a polycistronic mRNA and are often associated with related functions, molecular complexes or both. Such conserved gene blocks have been used in gene function prediction and phylogenetic analyses (Enault *et al.*, 2003; Overbeek *et al.*, 1999; Srinivasan *et al.*, 2005). There are several interesting biological questions that could be answered by studying the evolution of gene blocks: which components of the gene block tend to be more conserved? How did the gene block evolve? Given a gene block in a reference genome, which gene blocks from other taxa are homologous to it?

Annotating sequences of bacterial genomes and determining whether gene blocks are orthologous is essential for our understanding of bacterial genomes and their evolution. Recently, we have developed a heuristic method to identify novel orthologous gene blocks (Ream *et al.*, 2015). Motivated by this approach, we formalized the biological problem of finding orthologous gene blocks, which we name here the Relevant Gene Block problem. In this study, we show that the Relevant Gene Block problem is NP-hard. We then describe a greedy approximation method and assess its accuracy. Further, we formulate an Integer Linear Program for an exact solution suitable for conducting smaller studies and can serve as a baseline for evaluating the greedy method. We demonstrate the exceptional scalability and accuracy of our greedy method through extensive comparative empirical studies. From this point on, we will refer to the method in (Ream *et al.*, 2015) as the *Heuristic method*, to our method as the *Greedy method* and to the Integer Linear Programming formulation as the *ILP method*.

*Related Work:* several models have been proposed before to explain gene block and operon evolution. The models are not necessarily mutually exclusive, and different operons may evolve according to different models, or indeed a single operon may be the result of the combination of several models (Alm *et al.*, 2006; Bush *et al.*, 2018; Fani *et al.*, 2005; Goldberg *et al.*, 2016; Horowitz, 1945; Hsiao *et al.*, 2005; Koonin, 2009; Lawrence and Roth, 1996; Omelchenko *et al.*, 2003; Price *et al.*, 2006; Stahl and Murray, 1966). As mentioned earlier, in the previous work we developed the Heuristic method that explains the evolution of operons and orthoblocks as a combination of discrete events. In the course of their evolution, gene blocks may gain or lose genes, have genes duplicated or have them split away from the gene block. By determining the frequency of the events for any orthoblock in a clade, we can determine a cost for each event, and thus create a cost function to determine an optimal vertical path for the evolution of orthoblocks (Nguyen *et al.*, 2019; Ream *et al.*, 2015). We have also used the Heuristic method to vertically trace the evolution of operons on along a phylogenetic tree (Nguyen *et al.*, 2019), and discovered possible horizontal gene transfer events (Nett *et al.*, 2020).

*Contribution.* In this work we formalize the *event-based model* to define the essential *Relevant Gene Block (RGB)* problem. Given a reference gene block, and a set of homologous gene blocks in a target genome, this problem seeks to find orthologous gene blocks. We prove that this problem is inherently difficult, that is, NP-hard. Furthermore, we show that the RGB problem is unlikely to be efficiently approximated with a ratio bounded by a constant, meaning the problem is APX-hard. Despite these discouraging time complexity results, we describe an efficient greedy algorithm with an O(ln⁡n) approximation for the RGB problem. In addition, we provide an ILP formulation of the RGB problem suitable for smaller sized gene-block studies. Finally, in a comparative study of empirical data, we demonstrate our proposed methods’ outstanding performance in terms of scalability and optimization.

## 2 Preliminaries

### 2.1 Methods

First, we formalize the gene block evolution problem. To analyze the time complexity of this problem, we further formulate a relevant sub-problem, for which we demonstrate the NP-hardness and APX-hardness results. We then describe a greedy approximation algorithm for the gene block evolution problem. Finally, we provide an ILP formulation of the problem.

Here, we propose the *Relevant-Gene-Block problem* that is essential for analyzing the complex evolutionary histories of orthoblocks. In particular, we present the first mathematical formalization of the biological event-based model of orthoblock evolution.

### 2.2 The event-based model

A *reference taxon* is a taxon where operons have been reliably identified by experimental means. Such a taxon serves as a standard of truth to determine if the genes on a suspected orthoblock reside in an operon or a similar co-regulated gene block at least in one species. For this work, we chose *E.coli* K-12 MG1655 (NC 000913) as the reference taxon, since it belongs to the well-studied class of *γ*-proteobacteria in the Proteobacteria phylum.

**Fig. 1.**
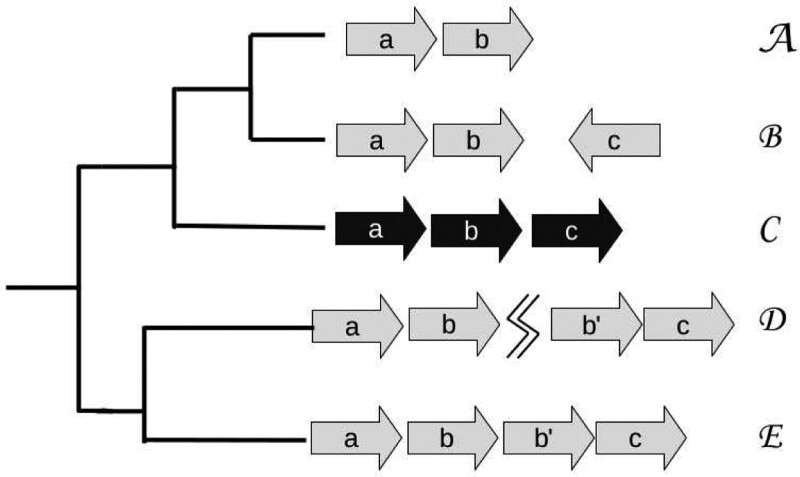
The orthoblocks from species *A* to *E* are arranged in a phylogenetic species tree. Species *C* has an experimentally determined operon (black arrows) and serves as the reference taxon. The orthologs in the species *A*, *B*, *D* and *E* were identified as described in (Ream *et al.*, 2015). The events between species *C* and all other species for this orthoblock are (1) *A*–*C*: deletion (of gene *c*), (2) *B*–*C*: split (of gene *c*), (3) *C*–*D*: duplication (of *b*) and (4) split (jagged line); *C*–*E*: duplication (of *b*)

An *event* is a single change in an orthoblock that is characterized as a *split*, *deletion* or *duplication*. Figure 1 depicts an example of such events. The *event-based cost* between any two orthoblocks is then defined to be the minimum possible number of splits, duplications and deletions required to explain the difference between them.

Given a reference operon O, we define $G:=\{x_{1},x_{2},x_{3},\ldots ,x_{n}\}$ to be the set of genes of O. A gene block B over G is a non-empty multiset of G defined as $B:=\{x_{1}^{\lambda_{1}},x_{2}^{\lambda_{2}},\ldots ,x_{n}^{\lambda_{n}}\}$ where $x_{i}\in G,\;\lambda_{i}\in \mathbb{N}$. We define the set of genes in gene block B as Gene(B) $:=\{x_{i}\;|\lambda_{i}\geq 1\}$, the duplication gene set of a gene block B as Dup(B) $:=\{x_{i}\;|\lambda_{i}\geq 2\}$, and the size of a gene block B as Size(B) $:=\sum \lambda_{i}$. An orthoblock *O* is a set of blocks that is either empty or contains at least one gene block of a size larger than or equal to two. We define the set of genes of *O* as $\text{Gene}(O):=\cup {}_{B\in O}$, and the set of genes that is duplicated in some gene blocks of *O* as $\text{Dup}(O):=\cup {}_{B\in O}$ Dup(B). Given a gene block B and a gene set *G* over G, we define $B\cap G:=\{x_{i}^{\lambda_{i}}\in B\;|x_{i}\in G\}$.

Costs for orthoblocks are described as pairwise functions that calculate the event count from one orthoblock to another, as a proxy for their evolutionary distance. The costs between any two orthoblocks *O* and O′ are defined as follows.

(1) The *split cost*, denoted as *c_s_*, is the absolute difference in the number of relevant gene blocks between the two taxa involved. We define Rel(O,O′) as the set of gene blocks from *O* where each gene in each gene block has to appear in O′ at least once. Formally, $\text{Rel}(O,O\prime ):=\underset{B\in O}{\cup }(B\cap \text{Gene}(O\prime ))$. The split cost can be formalized as:

(1) $$c_{s}(O,O\prime ):=\left|\left|\text{Rel}(O,O\prime )\right|-\left|\text{Rel}(O\prime ,O)\right|\right|$$

 

(2) $$=\left|\left|\underset{B\in O}{\cup }(B\cap \text{Gene}(O\prime ))\right|-\left|\underset{B\in O\prime }{\cup }\left(B\cap \text{Gene}(O)\right)\right|\right|.$$

If the orthoblock O′ is the reference gene block, the split cost between two orthoblocks *O* and *R* can be simplified as follows.

(3) $$c_{s}(R,O)=\left|\left|\text{Rel}(R,O)\right|-\left|\text{Rel}(O,R)\right|\right|$$

 

(4) $$=\left|\left|\underset{B\in R}{\cup }(B\cap \text{Gene}(O\prime ))\right|-\left|\underset{B\in O\prime }{\cup }\left(B\cap \text{Gene}(R)\right)\right|\right|$$

 

(5) $$=\left|\left|\underset{B\in R}{\cup }(B\cap \text{Gene}(O\prime ))\right|-\left|\underset{B\in O\prime }{\cup }(B\cap \text{Gene}(O\prime ))\right|\right|$$

 

(6) $$=\left|1-\left|\underset{B\in O\prime }{\cup }B\right|\right|=\left|1-\left|O\right|\right|=\left|O\right|-1.$$

For example, for the reference gene block R=(abcdefg), genome A has blocks O=((ab),(def)). We then compute the relevant gene blocks Rel(R,O)=(abdef) (removing genes *c*, *g*) and Rel(O,R)=((ab),(def)). Therefore, $c_{s}(O,O\prime )=|1-2|=1$.

(2) The *duplication cost, denoted as (c_u_)*, is the pairwise count of gene duplications between two orthoblocks. We define Dif(O,O′) to be the set of duplicated genes of gene block *O*, such that these genes also appear in O′ but are not duplicated in O′. Formally, Dif(O,O′):=(Dup(O)∩Gene(O′))∖Dup(O′). Here, our gene blocks are guaranteed to have at most one duplication of each gene for each block. We formalize the duplication cost as follows.

(7) $$c_{u}(O,O\prime ):=\left|\text{Dif}(O,O\prime )\right|+\left|\text{Dif}(O\prime ,O)\right|$$

 

=|(Dup(O)∩Gene(O′))∖Dup(O′)|

 

(8) 1+|(Dup(O′)∩Gene(O))∖Dup(O)|.

If the orthoblock O′ serves as the reference gene block, we can simplify the duplication cost between orthoblocks *O* and *R* as follows.

(9) $$c_{u}(R,O)=\left|\text{Dif}(R,O)\right|+\left|\text{Dif}(O,R)\right|$$

 

=|(Dup(R)∩Gene(O))∖Dup(O)|

 

(10) 1+|(Dup(O)∩Gene(R))∖Dup(R)|

 

(11) =|∅|+|(Dup(O)|=|(Dup(O)|

For example, for a reference gene block R=(abcde), the genome *A* has the gene block O=(abbcc). Observe that the orthologs of genes *O_b_* and *O_c_* are duplicated in genome *B*. We then compute Dif(R,O)=∅ and Dif(O,R)={b,c}. Therefore, $c_{u}(O,O\prime )=0+2=2$.

(3) The *deletion cost*, denoted as *c_d_*, is the difference in the number of orthologs that are in the orthoblocks of the genome of one taxon, or the other, but not in both. In other words, the deletion cost is the symmetric difference between the set of orthologous genes of the two gene blocks O,O′. We formalize the deletion cost as:

$$c_{d}(O,O\prime ):=|\text{Gene}(O)△\text{Gene}(O\prime )|.$$

If orthoblock O′ is the reference gene block, we can simplify the split cost between orthoblocks *O* and *R*
 

(12) $$c_{d}(R,O)=\left|\text{Gene}(R)△\text{Gene}(O)\right|$$

 

(13) =|Gene(R)|−|Gene(O)|asGene(O)⊆Gene(R).

For example, for a reference gene block R=(abcde), the genome *A* has gene block O=(abd) and the deletion cost between orthoblocks *R*, *O* is $c_{d}(R,O)=|\{a,b,c,d,e\}△\{a,b,d\}|=5-3=2.$

### 2.3 The Relevant-Gene-Block problem

Under the assumption that the reference gene block is the true operon, our problem is to identify the orthoblocks in our target genome so that the overall cost for the three events is minimized. Observe that this problem might be challenging since the event costs are not independent of each other in view of the fact that they contain the variable *Gene*(*O*). First, we describe a mathematical formalization of the problem, which we refer to as the *Relevant-Gene-Block (Deletion Duplication Split)* problem. Then we formulate restricted variations of this problem relevant for our time complexity analyses that involve a reduction from the minimum set-cover problem.

Problem 1. *[Relevant-Gene-Block (deletion, duplication, split)]*

*Instance:* <R,O>, *which are the reference gene block, and the set of homologous gene blocks in target genome.*

*Find:* <O′>  *where* O′⊆O, *so that* $c(R,O\prime ):=c_{d}(R,O\prime )+c_{s}(R,O\prime )+c_{u}(R,O\prime )$  *is minimum.*

Problem 2*. [Relevant-Gene-Block (deletion, split)]*

*Instance:* <R,O>, *which are the reference gene block, and the cluster of homologous gene in target genome.*

*Find:* <O′>  *where* O′⊆O, *so that* $c(R,O\prime ):=c_{d}(R,O\prime )+c_{s}(R,O\prime )=\left|\mathrm{Gene}(R)\right|-\left|\mathrm{Gene}(O\prime )\right|+\left|O\prime \right|-1$ is *minimum.*

Since *Gene*(*R*) is essentially the gene set of *R*, and one is a constant, we can reduce this problem further into its final form suitable for our analysis.

Problem 3*. [Relevant-Gene-Block (deletion, split, simplified)]*

*Instance:* <S,C>, *which are the set of genes of our reference block, and the collection of subset of the reference gene set respectively.*

*Find:* <O′>  *where* O′⊆O, *so that* f(S,O′):=|S|−|∪O′|+|O′|  *is minimum.*

We state the minimum set cover problem required for our reduction.

Problem 4*. [Minimum-Set-Cover]*

*Instance:* <S,C>, *which are the set of genes of our reference block, and the collection of subset of the reference gene set respectively.*

*Find:* <O′>  *where* O′⊆O, *so that* f(S,O′):=|S|−|∪O′|+|O′|  *is minimum.*

From now on, we use MSC to stand for the Minimum-Set-Cover problem, and RGB for Relevant-Gene-Block (deletion, split, simplified) problem.

## 3 Materials and methods

First, we prove the NP-hardness of the RGB problem by a reduction from the MSC problem. Our reduction relies on the essential property that the solution of the RGB problem should be a minimum set covering itself. Using this property, we introduce three lemmas that allow us to show the reduction. Second, we prove that our problem is equivalent in cost to the MSC problem and describe a greedy algorithm to provide an O(ln⁡n) approximation of the RGB problem. Finally, we describe an ILP formulation of the RBB problem.

### 3.1 Computational hardness of RGB

For the reduction, we are going to take an instance of the MSC problem and reduce it to an RGB instance that has a solution if and only if the MSC instance has a solution. Given an instance <S,C> of MSC where *S* is the set of ground elements and *C* is a collection of subset of *S*, we construct the same instance <S,C> for RGB problem, where *S* is the set of genes and *C* is the collection of subset of the reference gene set. Observe that ∪C=S.

Lemma 1. *If the set* C′  *is a solution to our RGB instance* <S,C>*, then* C′  *is the minimum set cover of the set* ∪C′.

Proof. Assume the contrary: $\exists C^{*}\subseteq C,\cup C^{*}=\cup C\prime ,|C^{*}|<|C\prime |$. We then have the following equality: $f(S,C^{*})=|S|-|\cup C^{*}|+|C^{*}|=|S|-|\cup C\prime |+|C^{*}|$. Since $\left|C^{*}|<|C\prime |,\;f(S,C^{*})<|S|-|\cup C\prime |+|C\prime |=f(S,C\prime \right)$. Hence, $f(S,C^{*})<f(S,C\prime )$, which contradicts that C′ is a solution to our RGB. Thus, C′ is the minimum set cover of the set ∪C′, as desired. □

Lemma 2. *If the set* C′  *is a solution to our RGB instance* <S,C>*, then* ∀c∈(C∖C′),|c∖C′|≤1.

Proof. Assume the contrary: $\exists c^{*}\in (C\setminus C\prime ),|c^{*}\setminus C\prime |\geq 2$. Consider $C^{*}=C\prime \cup \{c^{*}\}$. We then have the following equality: $f(S,C^{*})=|S|-|\cup C^{*}|+|C^{*}|=|S|-|\cup (C\prime \cup \{c^{*}\})|+|C\prime \cup \{c^{*}\}|=|S|-|\cup C\prime |-|c^{*}\setminus C\prime |+|C\prime |+1$.

Since $|c^{*}\setminus C\prime |\geq 2$, $f(S,C^{*})\leq |S|-|\cup C\prime |-2+|C\prime |+1=|S|-|\cup C\prime |+|C\prime |-1=f(S,C\prime )-1$.

Therefore, we have $f(S,C^{*})\leq f(S,C\prime )-1<f(S,C\prime )$. Hence, $f(S,C^{*})<f(S,C\prime )$, which contradicts that C′ is a solution to our RGB instance. Thus, ∀c∈(C∖C′),|c∖C′|≤1, as desired. □

Lemma 3. If the set C′ is a solution to our RGB instance $<S,C>,\;\forall c\in (C\setminus C\prime )\;\mathrm{and}\;|c\setminus C\prime |=1,C_{1}:=C\prime \cup \{c\}$ is the minimum set cover of $\cup (C\prime \cup \{c^{*}\})$

Proof. The proof is straightforward. As long as we add the subset that contributes exactly one to our cover so far, we increase the number of block by 1, and decrease the number of element to cover by 1. Therefore, $f(S,C_{1})$ does not change and is still the minimum. □

**Algorithm 1: Constructing solution of MSC given solution of RGB**

**Input**: *C*: a collection of subset, C′: a solution of RGB

**Output**: *C*_1_: solution of MSC C←C∖C′

$C_{1}\leftarrow C\prime$

**for**  c∈C  **do**

**if**  $c\setminus C_{1}==1$  **then**

$C_{1}\leftarrow C_{1}\cup \{c\}$

**return** *C*_1_

Claim 1. *If the set* C′  *is a solution to our RGB instance* <S,C>, *then we can construct the set C_1_ that is a solution to MSC instance* <S,C>  *using Algorithm 1 in polynomial time.*

Proof. Since C′ is a solution to the RGB instance <S,C>, according to Claim 1, C′ is a minimum set cover of the set ∪C′. By Claim 2, any subset *c* in the collection of subset *C*, but not in C′, can contribute at most 1 more element to our set C′. Hence, if we include all of the subsets *c* that contribute one extra element to our set *C*_1_ as in the for loop of Algorithm 1, we eventually cover all of the elements in the set *S*. Now, using Claim 3, our new set *C*_1_, after including each of such subsets *c* maintains the invariant that: *C*_1_ is the minimum set cover of $\cup C_{1}$. Therefore, *C*_1_ is a minimum set cover of *S*, and *C*_1_ is a solution for the MSC instance <S,C>. Observe that Algorithm 1 runs in polynomial time. Therefore, given that C′ is a solution to our RGB instance <S,C>, we can construct *C*_1_ that is a solution to the MSC instance <S,C> using Algorithm 1 in polynomial time. In addition, the solution to RGB has the same cost as the solution to MSC. □

Claim 2*. If the set* C′  *is a solution to our MSC instance* <S,C>, *then* C′  *is also a solution to RGB instance* <S,C>.

Proof. Since C′ is a solution to our MSC instance, we have f(S,C′)=|S|−|∪C′|+|C′|=0+|C′|=|C′|.

Assume the contrary, $\exists C^{*}\subseteq C,\;C^{*}$ is a solution to the RGB instance and $f(S,C^{*})<f(S,C\prime )$. Now, we can use Algorithm 1 to construct *C*_1_ that is a set cover of *S* and $f(S,C_{1})=f(S,C^{*})$ (from the Claims 1, 2 and 3). This provides the following property: $f(S,C_{1})=|S|-|\cup C_{1}|+|C_{1}|=|S|-|S|+|C_{1}|=|C_{1}|=f(S,C^{*}).$ Therefore, together with f(S,C′)=|C′| and $f(S,C^{*})<f(S,C\prime )$, we have $f(S,C^{*})<|C\prime |$. Since we have just proven that $f(S,C^{*})=|C_{1}|$, this means that $\left|C_{1}|<|C\prime \right|$ and *C*_1_ is a set cover of *S*. Hence, we can construct a set cover *C*_1_ that has size less than that of C′. This contradicts our assumption that C′ is a solution to our MSC instance (i.e. C′ is the minimum set cover of *S*). By proof of contradiction, C′ is also a solution to the RGB instance <S,C>. Furthermore, the solution to MSC has the same cost as the solution to RGB. □

Both, the NP-hardness and the APX-hardness of the RGB problem follow from the previous claims.

Theorem 1*. The RGB problem is NP-hard*

Proof. From the Claim 1 and Claim 2, we conclude that the MSC problem reduces in p-time to the RGB problem. Since the MSC problem is NP-Hard, it follows that the RGB problem is NP-Hard. □

Theorem 2. *RGB problem is APX-hard*

Proof. We observe that a solution of MSC and a solution of RGB are of equal in cost. Therefore, our reduction is also an approximation-preserving reduction. Henceforth, RGB is APX-Hard. □

### 3.2 Addressing the RGB problem

With the NP-hardness result in hand, it is unlikely that there exists an efficient algorithm that can optimally solve the RGB problem. We have also shown that the problem is APX-Hard, therefore RGB is unlikely to have an efficient approximation algorithm. However, our reduction shows that the solution of MSC problem has the same cost as the solution of RGB. Therefore, we devise a greedy algorithm for the RGB problem as follows.

#### 3.2.1 Greedy Algorithm

**Algorithm 2: Greedy Algorithm to solve RGB problem**

**Input**: *S*: gene set of the reference gene block, *C*: set of orthologous gene block

**Output**: C′: solution of RGB S′←∪C

C′←{}

**while**  S′≠∅  **do**

Select c∈C that maximizes |c∩S′|

S′←S′∖c

C′←C′∪c

**return**  C′

We will show that our algorithm is a ln⁡n approximation of our RGB problem. In order to do so, we need the following lemma.

Lemma 4. *Let C^opt^ be the optimal result of our RGB instance* <S,C>  *and* $C^{*}$  *be the optimal result of the MSC instance* <S′,C>  *as in Algorithm 2. We will show that:*
 

$$f(S,C^{\mathrm{opt}})=|S\setminus \cup C|+f(S\prime ,C^{*})$$

Proof. Following Lemma 1, *C^opt^* is the minimum set cover of $\cup C^{\mathrm{opt}}$. Then, using Algorithm 1, we can build a set *C*_1_ which is the minimum set cover of S′=∪C so that $f(S,C^{\mathrm{opt}})=f(S,C_{1})$. Hence, *C*_1_ is also an optimal result of the MSC instance <S′,C>, which means $\left|C_{1}|=|C^{*}\right|$. We expand the equality $f(S,C^{\mathrm{opt}})=f(S,C_{1})$ as follows:

$$f(S,C^{\mathrm{opt}})=f(S,C_{1})$$

 

$$=|S|-|\cup C_{1}|+|C_{1}|$$

 

$$=|S|-|\cup C|+|C_{1}|\text{as}\;C_{1}\;\text{covers}\;\cup C$$

 

$$=|S\setminus \cup C|+|C_{1}|$$

 

$$=|S\setminus \cup C|+|S\prime |-|\cup C^{*}|+|C_{1}|\text{as}\;C^{*}\;\text{covers}\;S\prime$$

 

$$=|S\setminus \cup C|+|S\prime |-|\cup C^{*}|+|C^{*}|\text{as}\;|C_{1}|=|C^{*}|$$

 

=|S∖∪C|+f(S′,C*)

Therefore, the proof is concluded. □

Theorem 3. *The greedy algorithm runs in polynomial time and provides an* O(ln⁡n)*—approximation of our RGB problem, where n is the cardinality of the set S.*

Proof. Firstly, we use the result from this paper (Chvatal, 1979). Given $C^{*}$ is the optimal solution to MSC of instance S′,C and C′ is the output of our alogrithm 2 we have:

$$f(S\prime ,C\prime )\leq (1+\ln n)f(S\prime ,C^{*})$$

 

$$↔f(S\prime ,C\prime )\leq (1+\ln n)(f(S,C^{\mathrm{opt}})-|S\setminus \cup C|)$$

 

$$↔f(S\prime ,C\prime )\leq (1+\ln n)(f(S,C^{\mathrm{opt}}-\delta ))$$

 

$$↔f(S\prime ,C\prime )\leq (1+\ln n)f(S,C^{\mathrm{opt}})$$

Let |S|=n,|C|=m, line 1 and 2 in the algorithm take linear time in *n*. Regarding the loop, each time we goes through the set of orthologous gene block to find the best gene block. Hence, the while loop takes *O*(*mn*). The claimed statement folllows. □

### 3.3 Integer linear programming

Given an instance *S*, *C* of the RGB problem, we define *x_i_* for every set $c_{i}\in C$ that takes a value of 1 if we include *c_i_* in our answer, and a value of 0 otherwise. We can express our RGB problem as the following integer linear program:

(14) $$\text{minimize}\sum_{i=1}^{n}x_{i}$$

 

(15) $$\text{subject}\;\text{to}\sum_{i,g\in c_{i}}x_{i}\geq 1\forall g\in \cup C$$

 

(16) $$x_{i}\in \{0,1\}\forall g\in S$$

The size of this version of ILP is the same as the size of ILP version of typical MSC problem, which is less or equal to |C|+|S|*|C|

Theorem 4. *Let C^opt^ be the optimal result of our RGB, and* $C^{*}$  *be the optimal result of the ILP formulation above. We can show that:*
 

$$f(S,C^{\mathrm{opt}})=|S\setminus \cup C|+f(S\prime ,C^{*})$$

Proof. Since $C^{*}$ is an optimal solution for ILP, it is also a solution to an instance $<S^{\prime },C>$ of MSC. Using Lemma 4, we can conclude the proof. □

## 4 Results

Here, we evaluate the performance of the Greedy method in approximating the RGB problem. The performance is analysed by comparing our algorithm with the previously best-known approach, the Heuristic method (Ream *et al.*, 2015) and our exact ILP solution as described in Section 4.3. All of our studies are performed using the previously used benchmark data-set (Ream *et al.*, 2015), which we describe first. In our comparative studies we first analyze the run-times (see Section 5.1), then the accuracy (see Section 5.2) and finally conclude the studies (see Section 5.3). We run all our experiment on a Dell Laptop, Intel Core i7 8th Gen, 15 GB DDR4 RAM running Ubuntu 18.04.4 LTS. Code was written in Python 3.5.

*Datasets* We used the experimentally identified operons from the *E.coli* K-12 genome as the gold standard. We chose *E.coli* because of the high quality of annotation, and the large number of experimentally verified operons. The genomes in which we looked for orthoblocks were taken from (Fani *et al.*, 2005). Previously the Heuristic method has been used on this group of taxa (Ream *et al.*, 2015), and the 55 operons were chosen based on the criteria: each operon comprised of at least 5 genes.

### 4.1 Scalability study

We compared the run-time of the Greedy method, the Heuristic method and the ILP approach, using datasets ranging from input sizes of 5 to 13 genes per operon.

**Experimental settings** We compare the running time of three methods. In each analysis, we ran the three methods on the same dataset (55 gene blocks) and recorded their running time in log ⁡10 (seconds).

**Fig. 2.**
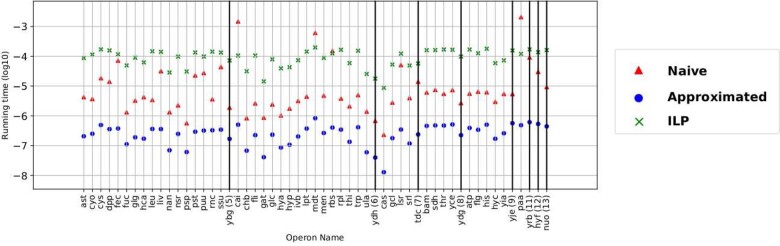
*X* axis: operon/gene block names, we sort the gene blocks in ascending order according to the number of genes in each block. From left to right: operon *ast* to operon *ygb* have 5 genes in the gene block; *cai* to *ydh* have 6 genes; *cas* to *tdc* have 7 genes; *bam* to *ydg* have 8 genes; *atp* to *yje* have 9 genes; *paa* to *yrb* have 11 genes, *hyf* has 12 genes. *nuo* has 13 genes. *Y*-axis: run time of the method in $\;{\text{log}}_{10}$ seconds

Overall, as shown in Figure 2, we can see that ILP takes the longest time to finish for all the operons except *cai, mdt, rbs* and *paa*. The Greedy method is the fastest without exception, beating the Heuristic method by a large margin. On average, the running time of the Greedy method is $2.9\times 10^{-7}$, of the Heuristic method is $8.8\times 10^{-5}$ and of the ILP method is $10^{-4}$. This means that the Greedy method should be around 100 times faster than the Heuristic method, and 1000 times faster than the ILP method. This is expected, since the Heuristic method tries to generate almost all possible combination of block, and the ILP method solves the problem exactly. To our surprise, the Heuristic method actually performs slowest in the four cases of *cai, mdt, rbs* and *paa*. The reason is not because those operons have more genes, but because they have more potential orthologous gene blocks. Although in the worst case the Heuristic method only takes milliseconds to finish, we only surveyed 55 operons within a group of 33 taxa. Currently, NCBI has more than 26 000 bacterial genomes. The running time will become important when running on more complex operons and larger number of species. In sum, the Greedy method performs the best in terms of run time.

### 4.2 Cost optimization

We evaluate the methods in terms of the event-based cost function. That is, we say that the method that is able to reconcile the blocks with the reference operon using the smallest number of split and deletion events is more accurate. The reasoning is that a lower cost is the most parsimonious explanation for the evolutionary distance between any two orthoblocks as explained in Nguyen *et al.* (2019). This is somewhat analogous the process of pairwise protein sequence alignment by assigning a cost function based on the costs of indels and substitutions.

**Fig. 3.**
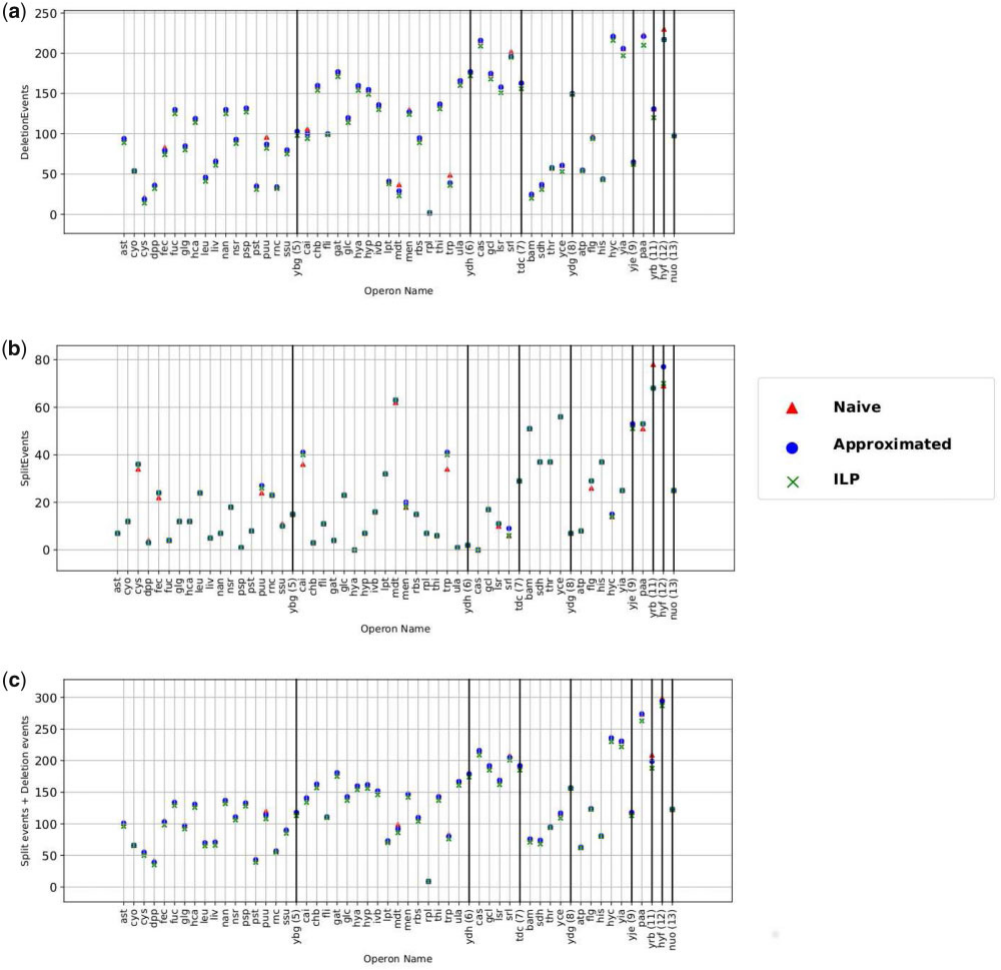
For *X*-axis values see Figure 2. The *y*-axis is the event count for the orthologous gene block in each species compare to the reference gene block. (**a**) Dots represents deletion events, (**b**) split events, (**c**) sum of deletion and split events

**Experimental settings** We generated the best orthologous gene block in each of the 33 species using three different methods. We then calculated the total number of deletion events and split events for each operon for each model and present it in Figure 3a and b. Then, for each model we calculated the sum of deletion events and split events for each operon (Fig. 3c) that represents our cost function. The method is considered better if the number of events is lower.

**Results and discussion** As shown in Figure 3a, in most cases the three models have the same number of deletion events. In 18 out of 55 cases, the Greedy method has a smaller number of deletion of events than the Heuristic method, and they have the same amount of deletion events. In all cases, ILP has the lowest number of deletion events. As shown in Figure 3b, the three models have the same number of split events in most cases. However, in 13 of 55 operons, the Heuristic method has a lower number of split events than the Greedy method. In 3 of 55 operons, the Greedy method has lower number of splits than the Heuristic method. Figure 3c conveys the cost function (sum of deletion and split events) for each of the methods. In 15 of 55 operons, the Greedy method has a lower cost than the Heuristic method. The Greedy and Heuristic methods perform similarly on the other operons. In all of the cases, the ILP method always has the lowest cost, as it is an exact method by design. Following that, the Greedy method is more accurate than the Heuristic method in 15 of 55 operons.

### 4.3 Concluding discussion

From our scalability and accuracy study we observe that the Greedy method is the fastest, being 100× faster than the heuristic method, and 1000× faster than the exact ILP method. In terms of accuracy, the Greedy method follows the ILP method very closely and either outperforms the Heuristic method or has the same results for all of the 55 operons. Therefore, on our small dataset that was used in (Ream *et al.*, 2015), our Greedy method is outperforming the Heuristic method in both scalability and accuracy.

## 5 Conclusions

Finding orthologous gene blocks is an important step in understanding the evolution of gene blocks and complexity in bacterial genomes. In this study we formally define the problem of identifying orthologous gene blocks given a reference operon, prove that it is NP-hard, and present an algorithm that guarantees O(ln⁡n) approximation and runs in polynomial time. In addition, we designed an ILP formulation and proved that it can solve our problem exactly. In our experimental study, we demonstrated that the Greedy method performs better than the Heuristic method in terms of both accuracy and scalability. We note that the methods developed in this work cannot handle the duplication events. While those are relatively rare, handling duplications would be an interesting topic for future work.
